# The Molecular Biology of Feline Immunodeficiency Virus (FIV)

**DOI:** 10.3390/v3112192

**Published:** 2011-11-09

**Authors:** Julia C. Kenyon, Andrew M. L. Lever

**Affiliations:** Department of Medicine, University of Cambridge, Box 157 Level 5 Addenbrooke’s Hospital, Hills Rd, Cambridge, CB2 0QQ, UK; E-Mail: jck33@mole.bio.cam.ac.uk

**Keywords:** FIV, retrovirus, lentivirus

## Abstract

Feline immunodeficiency virus (FIV) is widespread in feline populations and causes an AIDS-like illness in domestic cats. It is highly prevalent in several endangered feline species. In domestic cats FIV infection is a valuable small animal model for HIV infection. In recent years there has been sa significant increase in interest in FIV, in part to exploit this, but also because of the potential it has as a human gene therapy vector. Though much less studied than HIV there are many parallels in the replication of the two viruses, but also important differences and, despite their likely common origin, the viruses have in some cases used alternative strategies to overcome similar problems. Recent advances in understanding the structure and function of FIV RNA and proteins and their interactions has enhanced our knowledge of FIV replication significantly, however, there are still many gaps. This review summarizes our current knowledge of FIV molecular biology and its similarities with, and differences from, other lentiviruses.

## 1. Introduction

**Figure 1.**
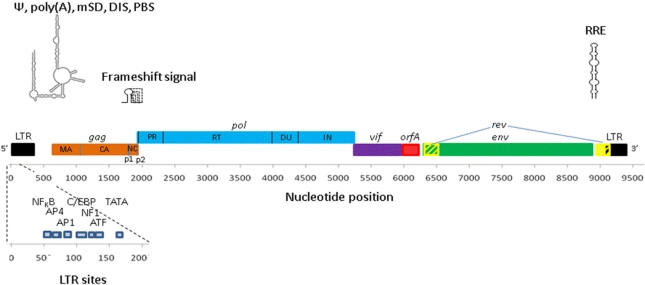
The organisation of the FIV genome. Genes and LTRs are shown roughly to scale*. pol* is translated from the full length RNA as a fusion Gag/Pol protein through a frameshift. LTR long terminal repeat; MA, matrix; CA, capsid; NC, nucleocapsid; PR, protease; RT, reverse transcriptase; DU, dUTPase; IN, integrase. *cis* acting RNA elements are shown above: ψ, packaging signal (RNA structure shown); mSD, major splice donor; DIS, dimerization initiation site; PBS, primer binding site; RRE, Rev response element. Regulatory elements are shown below in a magnified LTR.

**Figure 2.**
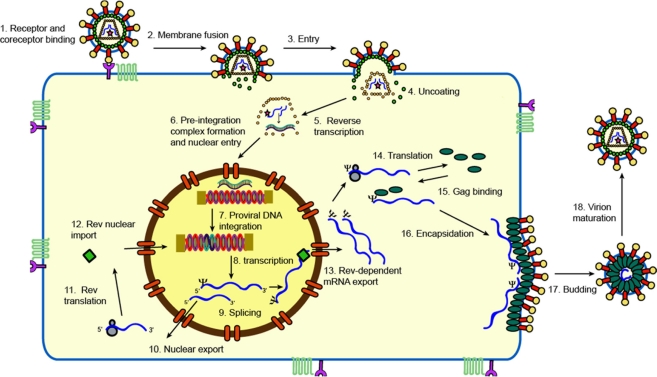
The feline immunodeficiency virus (FIV) replication cycle. Figure courtesy of Claire Williams.

Feline immunodeficiency virus (FIV) is a lentivirus infecting different species of cat and causing, in some, a pathology similar to that of HIV-1 infection in humans [1,2]. Species-specific strains of FIV circulate in many feline populations [3,4]. Domestic cats infected with FIV develop a progressive immune dysfunction characterised by depletion of CD4+ T cells; eventually signs of cachexia, gingivitis-stomatitis, wasting and neuropathological disorders appear, together with an increased incidence of malignancy [2,5–8]. Disease progression occurs over a similar time-scale to HIV-1 infection in humans. The severity of illness in the big cats is a matter of ongoing debate; some studies show little disease associated with FIV infection [9] while other studies in endangered big cat populations have identified lymphoid depletion and an AIDS-like illness [10,11]. Viral replication rate and the clinical symptoms seem to vary between different strains and comparisons of their genomic differences affords an insight into lentiviral molecular biology and pathogenesis [12]. Conversely, comparison of the well-researched gene products of the primate lentiviral genomes with those of FIV has allowed us a much greater understanding of FIV molecular biology. The structure of the FIV genome is illustrated in Figure 1. The FIV proteins and their functions are listed in Table 1. Here we summarise current knowledge pertaining to each phase of the FIV lifecycle (shown in Figure 2).

## 2. Entry

Lentiviruses target and enter cells by the viral envelope glycoprotein (Env) interacting with a target cell receptor and co-receptor. In the primate lentiviruses (human and simian immunodeficiency viruses HIV/SIV), the gp120 subunit of Env first binds to the primary receptor, CD4, which, due to its tissue distribution, targets HIV/SIV to helper T lymphocytes and cells of the monocyte/macrophage lineage. The initial interaction exposes a cryptic epitope on gp120 that enables interaction with a co-receptor (either CXCR4 or CCR5), leading to fusion of the cell membrane and viral envelope, and viral entry. Like the primate lentiviruses, FIV infects helper T cells and monocytes/macrophages, but through an initial interaction with the receptor CD134, rather than CD4 [13]. CD134 (also known as OX40) is a costimulatory molecule expressed most abundantly on activated feline lymphocytes and also on other lymphocytes, cells of the monocyte/macrophage lineage and dendritic cells [14]. Administration of blocking autoantibodies to CD134 interferes with FIV binding and confers improved host survival [15]. The cysteine-rich domains 1 and 2 (CRD1, CRD2) of feline CD134 are important for viral entry [16]. The structure of human CD134 is sufficiently different that it cannot support FIV entry [13], yet it is similar enough that replacement of CRD1 with its feline counterpart permits entry but with a lower efficiency [16]. To date, all domestic cat isolates of FIV tested have been found to use CD134 as a receptor and CXCR4 as a co-receptor; they do not interact with CCR5. Analogous to HIV, the interaction of FIV Env with CD134 exposes a cryptic epitope in the V3 loop of Env, that allows binding to CXCR4 [17]. The second extracellular loop of this chemokine receptor is crucial for viral entry during infection with both FIV and CXCR4-specific strains of HIV [18,19], suggesting that although the primary receptor is different, the mechanism of membrane fusion is likely to be similar in both viruses.

As lentiviral infection progresses, the virus comes under pressure from the immune system and mutates to escape neutralising antibodies. Env is shrouded in carbohydrate, linked to the protein via glycosylation sites, and this appears to be very important in masking potential neutralising epitopes; *de novo* mutations appear altering the glycosylation pattern in response to host antibodies [20]. A single region in the FIV envelope V5 loop plays a dominant role in whether the virus is neutralised or escapes neutralisation: a long-term study of viral variants during infection of individual cats found the appearance of many new *N*-glycosylation sites within this loop [21] and an insertion of just two residues at position 556–7 re-sensitises escape mutants to neutralisation [22]. Mutation of the other variable loops is common with the V1,V2 and V4 regions also showing variations in glycosylation pattern [21]. Pressure on the virus to mutate these variable loops can also lead to an alteration in the Env-receptor interactions. Advancing HIV-1 infection is often associated with a broadening cell tropism of the viral quasispecies. Co-receptor specificity of some of these species shifts from CCR5 to CXCR4, allowing the virus to infect different cell types. FIV seems only to use CXCR4 as a co-receptor, but, like HIV it shifts its cell tropism during the course of infection. This has been proposed to occur not by binding of a new co-receptor, but by a decreased dependence on CD134 (reviewed in [23]). Thus, as infection progresses, new Env variants are able to infect cells that express CXCR4 with limited amounts of CD134, such as naïve B cells and CD8 T cells [24,25]. Introduction of a lysine at residue 407 or 409 allows CXCR4 to be used as the sole receptor [26–28]; interestingly, these lysine residues have also been shown to be important for interaction with heparan sulfate proteoglycans [29], which play a role in the cellular attachment of some strains of both FIV and HIV [27,30]. Disruption of the glycosylation site at position 269 of the V1,V2 loop homolog is also thought to contribute towards CD134 independence [21,31]. Viruses that require CD134 as a primary receptor appear to bind to it via a complex interaction involving cysteine-rich domains 1 and 2, but as infection progresses the interaction becomes less complex and the interaction with CRD2 decreases [32]. Some researchers claim that the mutation rate of Env is low and have not observed these changes [33,34] however it is likely to be influenced by the replication rate of the FIV strain used.

Interestingly, some strains of lion FIV show a substantial divergence in structure and sequence of the envelope receptor binding domains and do not enter via CXCR4 and/or CD134 [35,36]. The cellular receptors for these subtype B lion FIVs have not yet been identified.

## 3. Reverse Transcription

After entry and uncoating, the single-stranded RNA genome is converted into double-stranded DNA by the process of reverse transcription. This commences from the tRNA primer, which is annealed to the primer binding site (PBS) of the viral genomic RNA. Extension from this primer towards the 5′end of the genome generates minus-strand strong-stop DNA. RNase H activity of the reverse transcriptase (RT) catalyses the removal of the RNA to leave single-stranded DNA. This new strand anneals via a complementary sequence to the 3′end of the genomic RNA where it acts as a primer for polymerisation of a DNA copy of the remainder of the RNA template. RNA fragments which resist RNase degradation are left at the polypurine tracts (PPTs) present in the centre of the genome (cPPT) and towards the 3′end (PPT) and act as primers for second strand synthesis. Polymerisation from the PPT halts at the first modified base of the primer tRNA and the product is known as plus-strand strong stop DNA. The RNase H domain of RT then degrades the tRNA and the 3′end of plus-strand strong stop DNA anneals to a complementary sequence at the 5′end of the minus-strand template to continue the plus strand and also allow completion of the minus-strand. This generates a double-stranded DNA provirus with long-terminal repeats and a central DNA flap at the cPPT. FIV RT utilises the same cellular tRNA primer as HIV-1 [37] yet it is unable to initiate reverse transcription on an HIV-1 template [38]. Reverse transcription initiation in lentiviruses seems to be regulated by an interaction between exposed nucleotides of the tRNA primer and part of the upstream genomic RNA (reviewed in [39]) however the sequences involved vary between viruses. In FIV initiation of reverse transcription is thought to be controlled by an interaction of the extreme 5′ nucleotides of the tRNA primer with a conserved stem-loop in the U5 region [38]. It is not known how progress of the RT through this stem-loop structure is governed but the block to reverse transcription in FIV appears to be less tightly controlled than in other retroviruses as a diverse range of retroviral RTs can extend efficiently on the FIV genome, whereas FIV RT could not extend on an HIV-1 genome unless the interaction between the tRNA primer and an A-rich upstream sequence was disrupted [37].

In FIV, unusually the two PPTs are different in sequence from one another. Unlike in HIV-1 and certain other retroviruses, the cPPT contains one or two pyrimidine residues, although it remains functionally similar to other cPPTs in initiating plus-strand synthesis and producing a DNA flap [40] which is suggested in HIV to be involved in nuclear import [41]. There is also the possibility that initiation from the central flap protects against G to A transition mutations caused by cytidine deaminase action during reverse transcription (discussed below, [42]).

FIV RT shares 63% nt identity and 67% amino acid similarity with HIV-1 RT and both are heterodimers of p51 and p66 subunits. The interactions between subunits are thought to be important for function and differ slightly between FIV and HIV as p51HIV-p66FIV and p51FIV-p66HIV chimeric RTs are less processive than their counterpart wild-type RTs [43]. Another slight structural difference is suggested by the fact that substitution into FIV RT of residues that contribute to NNRTI sensitivity in HIV-1 RT, does not render it drug susceptible [44] whereas the opposite chimera confers NNRTI resistance on HIV. Overall, though, FIV RT behaves in a very similar manner to other lentiviral RTs; utilising Mg^2+^ as a cofactor, able to function efficiently at the low dNTP concentrations present in non-dividing cells and possessing a similarly poor fidelity of replication that, with recombination, causes the high level of viral mutation [45,46].

Regulation of mutation rate is critical to lentiviral survival: it must be fast enough to escape the immune system, counteract restriction factors (and eventually to infect new hosts and species), but also constrained to prevent excessive mutation limiting production of viable viral particles. Like the non-primate lentiviruses CAEV, EIAV and Visna virus, FIV encodes a deoxyuridine triphosphatase (dUTPase) [47]. This enzyme reduces levels of dUTP by converting it to dUMP, a precursor for dTTP synthesis, and hence limits uracil misincorporation during reverse transcription. In dividing cells, cellular dUTPase levels are higher and dUTP levels are low, but in non-dividing cells the reverse is true and virally encoded enzymes are vital. dUTPase- FIV mutants, like their EIAV counterparts, are able to productively infect dividing cells at or near wild-type levels, but are unable to replicate in primary macrophages [47,48]. When FIV dUTPase deletion mutants are used to infect domestic cats, they are able to establish a productive infection, but the virus burden is reduced and the genomic mutation rate is five times greater in primary macrophages, which are non-dividing, than in actively dividing T cells. This leads to changes in the tissue distribution of the virus [49]. The dominant mutations are G to A transitions, consistent with uracil misincorporation [49]. The FIV dUTPase is remarkably similar in structure and mechanism to the *E.coli* dUTPase [47] and is likewise active as a trimer [50]. The presence of a dUTPase in other parts of the genome of type B and D retroviruses suggests that it may have been acquired horizontally [51–53]. Interestingly, primate lentiviruses do not encode a dUTPase although there is evidence that an ancestral version may have evolved into part of the gp120 envelope gene [54,55]. Their strategy to limit uracil misincorporation is not yet clear, although it is possible that HIV-1 achieves this by an association between the Vpr protein and uracil *N*-glycosylase, which removes misincorporated uracil [56].

The genome may also mutate during reverse transcription by the action of host cell APOBEC3 proteins. These are a family of cytidine deaminases which act upon single stranded DNA during reverse transcription and convert cytosine to uracil, introducing a G to A mutation in the viral RNA. Different members of the APOBEC3 family have been shown to act against retroviral infection; in feline cells APOBEC3H and APOBEC3CH proteins mutate the FIV genome, and are counteracted by the viral Vif protein [57]. HIV-1 Vif targets the APOBEC3 protein to an E3 ubiquitin ligase complex, leading to polyubiquitination and degradation by the proteasome. This is thought to occur via an interaction with Elongin C (EloC), part of an E3 ligase complex. The anti-APOBEC3 activity of FIV Vif is dependent on an EloC interacting motif with the sequence TLQRLA, suggesting that it uses an analogous mechanism to that of HIV-1 [58]. However, their mechanisms are not similar enough to be able to substitute for one another, as FIV Vif is unable to counteract human APOBEC3 proteins [58]. Vif is crucial to FIV and HIV replication *in vivo*, as viruses in which Vif is deleted barely replicate [59,60].

## 4. Integration

The reverse transcribed genome is part of a nucleoprotein structure known as the pre-integration complex (PIC) which is translocated to the nucleus where the viral genome is integrated into the host cell DNA. A factor known as LEDGF/p75 (Lens Epithelium Derived Growth Factor), a host cell factor whose putative role is in transcriptional coactivation, interacts directly with lentiviral integrases and is thought to be responsible for tethering the viral integrase (IN) to host cell chromatin [61]. The interaction is specific to lentiviruses [61,62] and there is a suggestion that LEDGF/p75 may protect FIV (and HIV) IN from proteasomal degradation [63]. FIV IN doesn’t contain a nuclear localization signal (NLS) but, like HIV-1 IN, localizes to the nucleus when LEDGF/p75 is present and is seen in the cytoplasm when it isn’t [64]. Although other proteins, such as transportin-3 (TNPO3) have been proposed as nuclear import shuttles for the PIC, FIV IN appears to be at best minimally dependent on it for nuclear import [65], and the mechanism remains elusive, although it may depend on integrase multimerisation [66].

Multimerisation of the integrase *is* important for its catalytic functions [67]. The N-terminus chelates zinc which induces multimerisation and increases catalytic activity [68–72]. Integration proceeds in three steps: firstly, the 3′ ends of the viral DNA are processed to remove two nucleotides and leave CA overhangs. The 3′ ends are then joined to the 5′ ends of cleaved target DNA, leaving a gapped intermediate. Finally the gaps are repaired and the 5′ ends are ligated. Like HIV-1 IN, FIV IN uses Mg^2+^ or Mn^2+^ as a cofactor, and contains 3 domains: N and C terminal domains which are needed for 3′end processing and joining, and a catalytic domain in the core (CCD) [73]. The predicted three dimensional structure of the FIV IN CCD is almost identical to that of HIV-1 [74] and despite lower sequence similarity across the rest of IN and other proteins, phylogenetic analysis places the FIV CCD as the closest relative of HIV and SIV CCDs. Lending support to the structural and phylogenetic findings, integrase strand transfer inhibitors (INSTIs) appear to block FIV replication as efficiently as HIV-1 replication [74]. However, FIV displays a slightly different integration site preference from that of HIV-1 [75,76].

## 5. Transcription and Nuclear Export

Transcription of the HIV-1 genome is transactivated by binding of the viral Tat protein to the TAR loop in the nascent RNA strand at the 5′ end of the genome. FIV contains no predicted TAR-like structure. It contains an accessory gene, OrfA (also known as Orf2) with limited sequence similarity to Tat which has been shown to transactivate transcription from the FIV LTR to varying degrees [77–79]. This protein does not bind directly to the FIV LTR but has been suggested to act in conjunction with unidentified cellular transcription factors [79]. The FIV LTR contains known *cis*-acting elements including binding sites for AP1, AP4, C/EBP, NF1 and ATF [80–83] of which the AP4/AP1 and ATF sites appear to be required for full basal promoter activity [77,84–86]. The AP1, C/EBP and ATF motifs have been suggested to co-operate to enhance transcriptional activity in conjunction with OrfA. Basal promoter activity in FIV is similar to that of CAEV and Visna virus, and is significantly higher than for HIV [87,88]. Visna and CAEV Tat proteins contain an important cluster of Cys residues at their C-termini [89], and when a homologous region in FIV OrfA was deleted, transactivation of the FIV LTR was blocked, suggesting that these three viruses use similar methods of transcriptional control [79].

Lentiviral transcripts contain multiple splice sites for qualitative and quantitative control of expression of the various genes products. Control of splicing is therefore important to the lentiviral lifecycle and changes in regulation of exonic and intronic splicing silencers (ESS and ISS) and other protein families involved in splicing regulation have been shown to occur during HIV-1 infection of CD4+ T cells [90]. The ESS and ISS families bind to sites within exons and introns respectively and reduce the likelihood of nearby sites being used as a splice junction. FIV OrfA has been shown by microarray analysis to downregulate expression of hnRNPA, hnRNPA2B1 and hnRNPH [91]; proteins that regulate ESSs and ISSs, alongside 4 SR proteins that are required for the selection of alternative splice sites and removal of constitutively spliced introns [91]. Three snRNP subunits of the spliceosome were also found to be modulated by the presence of OrfA [91].

A consequence of encoding multiple splice sites within one transcript is the presence of introns which would normally prevent nuclear export. Lentiviruses encode a Rev protein, expressed from a fully-spliced viral mRNA. Rev shuttles back into the nucleus to allow export of partially spliced and unspliced viral transcripts by binding to the Rev-response element (RRE), a highly structured part of the RNA genome, and to the nuclear export protein CRM-1 (exportin-1), which shuttles Rev and its target RNA out of the nucleus. Unlike the primate lentiviruses, but similar to CAEV and BIV, the first coding exon of FIV *rev* overlaps *env* and is in the same open reading frame [92–94]. HIV-1 Rev has two functional domains: the first contains an arginine-rich region thought to recognize and bind to the RNA of the RRE, and a nuclear localization signal [95]. There is sequence similarity between this domain in HIV-1 and FIV [96]. The C-terminal domain contains the nuclear export signal [97]. In the primate lentiviruses this is a conserved 11 amino acid, leucine rich region that mediates binding to CRM-1, but in FIV the region is longer, and the leucines are spaced further apart and are interspersed with other hydrophobic residues [98]. This has since been identified as part of a family of Crm-1 dependent nuclear export signals including that of GAPDH and human U5snRNP [99], and can substitute for the HIV-1 Rev leucine-rich domain [100]. In the primate lentiviruses the RRE overlaps the middle of the *env* ORF, making mutagenesis of the RRE difficult without disrupting Env function, but in FIV the RRE resides at the very 3′end of the *env* ORF, making mutagenic studies possible. The RRE folds into a conserved stem-loop of around 150 nts and it appears that the entire structure except for the very tip (stem 6 and the loop) is critical for Rev function: structural perturbation of any of the other 5 stems disrupts FIV replication [101].

## 6. Packaging and Assembly

Translation commences from the fully spliced Rev transcript, and increasing Rev concentration allows the transport and translation of the partially spliced and unspliced (genomic) RNAs. Retroviral Gag proteins are translated from the genomic transcript and perform multiple functions according to the stage of the viral lifecycle. Inside the host cell, the Gag protein identifies and captures the viral genomic RNA for packaging, isolating it from the wealth of other RNAs present in the cytoplasm [102]. Although the cellular location for this interaction is unknown in any retrovirus, recent experiments in FIV suggest that it may occur at the cytoplasmic face of the nuclear envelope [103]. The stringent specificity of Gag for the viral genomic RNA is mediated by the nucleocapsid (NC) domain, which recognizes within it one or more packaging signals (Ψ). In some retroviruses Ψ is positioned between the major splice donor (mSD) and the Gag AUG, and thus is retained only in genomic and not in spliced viral RNAs. In FIV Ψ appears to be bipartite, lying partly within the first 250 [104] nt of the 5′UTR, and partly in the start of the *gag* gene [104–107]. Intriguingly, the intervening region, containing the mSD through to the Gag AUG, is neither structurally nor functionally important as it can be deleted or replaced with heterologous sequence without affecting packaging [108]. Although there is little sequence conservation between these regions of FIV and other lentiviruses, the recognition of viral genomic RNA must occur via similar mechanisms as FIV Gag can cross-package HIV-1 and SIV Ψ-containing RNA, and vice-versa [109]. The lack of sequence similarity between these viruses suggests that the structural context of these binding sites is very important. Genomic RNA dimerization is closely linked to packaging and the *in vivo* dimerization initiation site in FIV has not been identified, but *in vitro* a palindromic sequence within the gag ORF initiates dimerization [110]. How the virus regulates the balance between translation and packaging of its genomic RNA is not clear, however there is evidence from biochemical probing, free energy minimization and phylogenetic comparisons that the genomic RNA structure can adopt two different conformations[110]. In one of these the putative dimerization initiation site is exposed suggesting it may favour RNA packaging. The ability to adopt either structure is highly conserved amongst FIV domestic cat isolates[110].

The RNA is captured by a high affinity interaction between the NC domain of the Gag polyprotein and the RNA, but it is likely that the entire Gag protein plays a role in Ψ recognition and binding. NC contains two Zinc knuckle domains which are conserved amongst lentiviruses and most other retroviruses, and contain the sequence Cys-X2-Cys-X4-His-X4-Cys. The proximal domain plays a more dominant role in binding to the FIV genome than the distal domain and two lysine residues in the linker region between the Zn knuckle domains are also crucial [111]. In FIV, the two zinc knuckles have also been observed to contribute towards nucleic acid annealing, minus strand DNA transfer, the level of dimerization seen, hybridization of tRNA to the PBS, and initiation of reverse transcription [112].

With the exception of some highly conserved motifs, the overall sequences of Gag proteins are highly variable between lentiviruses. Interestingly, the MA protein sequence appears much less conserved than CA or NC. This divergence is maintained even within different strains of FIV, as the Gag proteins from different species-specific strains of FIV vary by 25–30% [113]. However, structural predictions of FIV Gag suggest that the protein adopts a similar structure to that of HIV-1 and EIAV Gag proteins [113], with MA, CA and NC domains present in similar numbers and with similar arrangements of secondary structural elements.

Retroviral Gag proteins comprise the main structural components of virions, and Gag alone self-assembles *in vitro* into virus-like particles. The appearance of these is similar in FIV to other lentiviruses [114,115]. The CA domain is known to facilitate Gag-Gag interactions via a dimerization interface, and cross-reactivity of antibodies raised against FIV or other lentiviral CA proteins suggests that although sequence conservation is low, they are likely to be structurally similar [116,117]. A Pro-Asp salt bridge that is involved in stabilizing CA-CA interactions in other retroviruses is predicted to form in all FIV strains [113], and the presence of a conserved major homology region (MHR) also suggests that CA-CA interactions develop in a similar manner to that of HIV-1 [118,119].

Another function of Gag is the formation and budding of the developing virion. Several motifs are important for this purpose; as is the case for most retroviral matrix (MA) domains, myristylation of the N-terminus of FIV MA enables its association with the lipid bilayer [115]. The interaction of a patch of basic residues in MA with the plasma membrane component phosphatidylinositol-4,5-bisphosphate (PIP_2_) is also thought to be important for viral egress, as perturbation of PIP_2_ levels affects budding [120]. It is possible that this interaction is weaker in FIV than in other lentiviruses as FIV MA contains a shortened basic motif and is incapable of substituting for SIV MA unless the basic patch is mutated to include two critical lysine residues found in SIV (and HIV-1), although SIV MA can substitute for FIV MA without affecting viral replication [121]. A patch of hydrophobic residues, near the N-terminus that mediates membrane interactions in other retroviruses [122,123], is dispensable in FIV [115].

Retroviral Gag proteins contain “late” domains which are short motifs that interact with the cellular transport and budding machinery and promote viral egress. The PSAP motif in the FIV Gag C-terminal peptide p2 is highly conserved and, like the PTAP motif in HIV-1, interacts directly with the ESCRT machinery through Tsg101 [124]. This is vital for viral budding and virion morphogenesis and maturation [111,124]. Although the overall process is similar there are variations in each retrovirus. The N-terminal proline in the FIV PSAP motif does not seem to be involved in late domain function unlike in HIV-1 where it is critical [125,126], and human Tsg101 interacts more strongly with residues surrounding it than it does with that of HIV-1 [124]. Other late domain interactions in FIV are less well understood. The ESCRT-associated protein Alix facilitates virion release in HIV-1 and EIAV via its interaction with a YPXL motif, but although an initial study found the similar LxxL motif in FIV to be essential [111], in a subsequent study mutation in the motif seemed to have no effect on viral morphogenesis [124]. The third type of known retroviral late domain is PPxY, which interacts with E3 ubiquitin ligases such as Nedd4 to promote ubiquitination of Gag. Ubiquitination is thought to enhance interactions with the ESCRT machinery and hence promote viral egress, by unknown mechanisms. Although no PPxY motif is present in FIV Gag, it is ubiquitinated by Nedd4-like proteins, and this can compensate for mutations in the PT/SAP motif [126]. However, neither the molecular details of this nor its relevance during FIV infection is known.

Gag also binds to Env and incorporates it into virions. HIV-1 Env binds MA via an interaction of MA, TIP47 protein and the cytoplasmic tail of Env, although whether an analogous mechanism exists in FIV has not been identified [127].

Budding of enveloped viruses is prevented in human cells by tetherin: a transmembrane protein that anchors the budding virion to the cell surface [128]. Lentiviruses have evolved different ways in which to counteract tetherin, suggesting that it is indeed a retroviral restriction factor; these functions are encoded by HIV-1 Vpu, HIV-2 Nef and HIV-2 and SIV Env proteins [128–132]. Feline cells also express tetherin in response to viral infection; it is able to prevent release of FIV and HIV-1 from the cell surface, but intriguingly, does not prevent cell-to-cell spread of the virus, and in the case of a CD134-independent strain of FIV, it enhanced syncytial formation [133]. *In vivo*, this may allow the selective expansion of CD134-independent viral variants and promote more efficient cell-to-cell spread [133]. Unlike the primate lentiviruses, FIV does not appear to contain a functional tetherin antagonist [133], suggesting that tetherin may not be an FIV restriction factor and that conversely FIV may have evolved to use tetherin to its advantage.

OrfA has recently been shown to downregulate CD134 on the surfaces of infected cells [134]; this is thought to aid the dissemination of budding virions by minimizing their interactions with the surface of the infected cell.

## 7. Maturation

During virion maturation the cleavage of Gag and Gag-Pol precursor polyproteins is performed by the viral protease (PR). In the Gag-Pol polyprotein the protease is nestled between p2 and RT and is monomeric, but it dimerises during budding. PR then cleaves 9 separate proteins from the polyprotein precursors: MA, CA, p1, NC, p2, PR, RT, RNase H, dUTPase and IN [135]. The order of this cleavage is very important to the formation of a viable virion, and protease inhibitors often function by interfering with this [136]. In FIV the cleavage sites are positioned similarly to HIV-1, but are different in both amino acid and processing sequence, reflecting a difference in cleavage site preference of their PRs [137]. In addition, FIV has a dUTPase to cleave and has fewer spacer peptides. The process of cleavage leads to virion maturation and activation of some of the enzymes, which allows the virion to infect a new cell. Autoproteolysis of PR in FIV and other retroviruses also regulates its own activity [138]. Like HIV-1, FIV PR contains 4 autoproteolysis sites; however, after processing of the polyprotein the mature protease is 116 amino acids, whereas in HIV it is 99 amino acids. Despite this length difference, and the fact that FIV and HIV-1 PRs share only 23% aa identity, the three-dimensional structures are strikingly similar, as they are in all retroviral proteases [139–141]. The structures follow a template of repeating secondary structural units: A hairpin with loops (A1 and A2), a wide loop (B1, containing the catalytic aspartic acid and B2), alpha-helices (C1 and C2) and a second hairpin or B-strand (D1, D2). The loop regions and connections between secondary structural elements vary in length and are the main source of structural difference between the retroviral proteases (reviewed in [141]). The substrate specificities and the susceptibilities to protease inhibitor drugs vary between FIV and HIV-1, and the residues in the active site have been observed to be different. FIV prefers Ser at P1’, and Asn/Gln at P1 and Arg at P3, and HIV prefers Asn at P2 and Glu/Gln at P2 [142]. Mutation of the FIV active site to include the analogous HIV-1 residues alters the order of Gag-Pol processing to more closely resemble that of HIV-1 and increases sensitivity to HIV-1 protease inhibitors [137]. Such chimeric comparison studies are possible because the three-dimensional structures of the enzymes are so similar. It is hoped that such studies will lead to the development of broad-based inhibitors against retroviral protease function, that are active against proteases resistant to current drugs and that prevent the emergence of drug escape mutants.

## 8. Conclusions

Despite significant sequence divergence from the primate lentiviruses, the molecular biology of FIV is strikingly similar. The structures and mechanisms of action of the essential proteins are very closely related in most cases, and result in a similar cycle of infection and morphologically indistinguishable virions. Differences in genomic organisation in FIV from other lentiviruses, such as the location of the RRE, and the lack of overlap of the late domains of Gag with the *pol* ORF have facilitated delineation of the functions of these various elements. However, many aspects of the replication cycle are still obscure. In particular, the role of OrfA and its involvement in both transcriptional transactivation and splicing regulation remains controversial. Aside from the functions discussed above, it was seen to downregulate several E2 ubiquitin conjugating enzymes and one ubiquitin-protein ligase [91]. OrfA may therefore limit degradation of viral proteins in the host cell. It may also interfere with antigen processing as it downregulates two subunits of the immunoproteasome [91]. It will be very interesting to see how such a small protein can have such multifaceted effects on the host cell and the virus. The similarities in clinical course of infection between domestic cat FIV and HIV-1 infection are striking and many have proposed the use of FIV as a small-animal model for HIV-1 infection. The similarities in molecular biology between the two viruses add weight to this argument. Interestingly, aside from the entry receptor, HIV-1 needs only FIV Vif in order to replicate in feline cells [143], which indicates that all of the interactions between HIV-1 and feline cellular components are functional, and again highlights the striking similarities in their molecular biology. In remarkable contrast, Vif from domestic cat strains of FIV can counteract APOBEC proteins from puma, lion, tiger and lynx, but cross-species infection is unproductive, suggesting that in the cells of these animals further restrictions to infection with different species-specific FIV strains exist [144] highlighting the differences between the different feline species and their individual FIV strains.

## Figures and Tables

**Table 1.** Major functions of the FIV proteins.

**Protein Precursor**	**Functional Cleavage Products**	**Major Attributed Functions**
Gag	Matrix	Virion structural protein
	Capsid	Virion structural protein
	Nucleocapsid	Binding to viral genome
Pol	Protease	Cleavage of Gag and Gag-Pol precursor proteins, leading to virion maturation
	Reverse Transcriptase	Reverse transcription of the genomic RNA into proviral DNA
	Deoxyuridine triphosphatase	Limitation of uracil misincorporation during reverse transcription
	Integrase	Integration of proviral DNA into host chromosome
Vif		Counteraction of host cell cytidine deaminases
OrfA		Possible roles in transcriptional activation, control of splicing, virion dissemination
Rev		Nuclear export of partially spliced /unspliced RNA transcripts
Env	Surface and transmembrane subunits	Virion attachment and entry into target cells
